# Obesity marker trajectories and cognitive impairment in older adults: a 10-year follow-up in Taichung community health study for elders

**DOI:** 10.1186/s12888-022-04420-1

**Published:** 2022-11-30

**Authors:** Tsai-Chung Li, Chia-Ing Li, Chiu-Shong Liu, Chih-Hsueh Lin, Shing-Yu Yang, Cheng-Chieh Lin

**Affiliations:** 1 Department of Public Health, College of Public Health, China Medical University, Taichung, Taiwan; 2 Department of Healthcare Administration, College of Medical and Health Science, Asia University, Taichung, Taiwan; 3 School of Medicine, College of Medicine, China Medical University, No. 100, Sec. 1, Jingmao Rd., Beitun Dist, Taichung, 406040 Taiwan; 4 Department of Medical Research, China Medical University Hospital, Taichung, Taiwan; 5 Department of Family Medicine, China Medical University Hospital, Taichung, Taiwan

**Keywords:** Fat mass, Abdominal fat, Trajectory, Cognitive impairment, Cognitive decline

## Abstract

**Background:**

Obesity and cognitive impairment prevalence increases as age increases. Recent growing evidence finds links between obesity and cognitive impairment in older adults. However, the association between the two is controversial. This study aims to identify obesity marker trajectory patterns, and to assess whether these patterns are associated with cognitive impairment and cognitive decline during a 10-year follow-up period among community-dwelling older adults.

**Methods:**

A total of 626 older adults aged 65 and older were involved in the study, with at least two repeated measurements at baseline, one-year or 10-year follow-up. Cognitive function was measured through the Mini Mental State Examination. Obesity markers included body mass index, waist circumference, waist-to-hip (WHR), fat mass (FM), and abdominal fat (AF) measured by dual-energy X-ray absorptiometry. Multivariate logistic regression analyses were performed to estimate the adjusted odds ratios (ORs) and 95% confidence intervals (CIs) of cognitive impairment and cognitive decline for obesity marker trajectory patterns.

**Results:**

After a 10-year follow-up, 168 older adults with incident cognitive impairment and 156 with rapid cognitive decline were defined as the top 25th percentile of cognitive decline. Four distinct trajectory groups of obesity markers were identified. In multivariate logistic regression analyses, a low likelihood of cognitive impairment was observed in the consistently high-level group from FM trajectory (ORs = 0.41, 95% CI = 0.20–0.85); the high-level U-shaped group from WHR trajectory (0.43, 0.22–0.84); and the median-level flat inverse U-shaped, consistently high-level, and low-level flat U-shaped groups from AF trajectory (0.44, 0.26–0.77; 0.33, 0.18–0.61; 0.39, 0.18–0.82). In addition, a low likelihood of rapid decline was found in the low-level, slightly increasing trend group from WHR trajectory (0.43, 0.22–0.85).

**Conclusion:**

FM and AF trajectories with consistent high levels and WHR trajectory with high level with U-shaped group are associated with low risks of incident cognitive impairment in older adults. Similarly, WHR trajectory with a low but slowly increasing trend is associated with a decreased risk of cognitive decline.

**Supplementary Information:**

The online version contains supplementary material available at 10.1186/s12888-022-04420-1.

## Background

Approximately 9.3% of people worldwide (727 million) were aged 65 or older in 2010, and it is expected to rise by 16.0%, reaching 1.5 billion, by 2030 [1]. Taiwan has one of the fastest aging populations in the world, and aging problem is becoming an imperative issue [2]. Cognitive impairment and dementia prevalence and incidence increase as the population ages and have become a huge economic burden to the whole society. The prevalence in elderly people is 18.9% for mild cognitive impairment (MCI) using the expanded Mayo Clinic criteria [3] and 5–10% for dementia in high-income countries [4]. MCI is considered an intermediate phase from normal aging to dementia [5]. Given that treatment options for dementia are limited, identifying factors that can prevent or delay age-related cognitive decline as a strategy to reduce dementia is important [6]. Therefore, understanding potential modifiable risk factors for cognitive impairment or cognitive decline is necessary.

Obesity and overweight prevalence have been rising, even among older persons. Many studies have examined the associations of obesity markers, such as overall obesity markers of body mass index (BMI) [7–18] and fat mass (FM) [9, 17–19], and central obesity markers of waist circumference (WC) [8, 13, 14, 18], waist-to-hip ratio (WHR) [12–15, 17, 18, 20], abdominal fat (AF) [9, 18], and visceral fat [14] with cognitive function. The body composition measurements of FM, AF, and visceral fat were either assessed by gold standard approach of dual​-energy X-ray absorptiometry (DXA) scan [9, 17] or a rapid, non-invasive approach of bioimpedance analysis (BIA) [14, 18, 19]. As for cognitive function, most of studies adopted global cognitive function [7, 10–15, 17–20] to define severity of dementia [7], cognitive impairment [10–13, 15–19], cognitive decline [14], or cognitive performance [20]. The commonly-used screening tool was mini-mental state examination (MMSE) [7, 11–13, 15, 16, 19]. Domain-specific cognitive function was assessed in one study using comprehensive neuropsychological assessment batteries [9].

Most of these mentioned above studies are cross-sectional associations [7–19]. Two prior studies report that sex differences exist in the cross-sectional relationship between obesity and MCI among older adults [11, 17]. However, determining whether obesity transition is a predictor of cognitive impairment and cognitive decline within the elderly population using a cohort study design remains controversial [20, 21]. The Northern Manhattan Study [20] used baseline overall obesity (measured by BMI) or abdominal obesity (measured by WHR) to assess their relationship with global cognitive performance at subsequent assessment and cognitive changes over time among older populations, but no associations were found. The findings of a nationwide retrospective cohort study conducted in China indicates a higher level of BMI at baseline and larger WC are associated with a slower rate of global cognitive decline whereas greater BMI variability was associated with a faster rate of global cognition score decline [21]. Due to inconsistent results, evaluating the effects of obesity markers with cognitive function in older adults is needed. In addition, overall obesity marker of FM and central obesity marker of AF have not been assessed in these two prior studies.

Previous studies found that overall obesity (defined by BMI or FM) [7–12, 17–19, 21] and abdominal obesity (defined by WC, WHR or AF) [12–16, 18, 21] can predict cognitive impairment, but prior research exploring the joint effects of these obesity factors is scarce [13]. Only one study indicates that overall obesity, measured by BMI, and central obesity, measured by WC or WHR, have combined effects exerting a significant increase in cognitive impairment prevalence more than in that with BMI overweight/obesity alone [13]. Therefore, our specific objectives are to evaluate the independent and joint effects of overall obesity trajectories, measured by BMI or FM, and abdominal obesity trajectories, measured by WHR, WC, and AF, on cognitive impairment and cognitive decline in elders who participated in the Taichung Community Health Study for Elders (TCHS-E). Based on prior studies’ findings [7–19, 21], we hypothesized that obesity markers with increasing trend of trajectories would be associated with lower likelihoods of cognitive decline or impairment.

## Method

### Study design and subjects

A community-based prospective cohort study, namely, TCHS-E, was conducted in 3997 residents aged 65 and over in the North District of Taichung City, Taiwan in 2009. All participants were invited to join by letter, phone, and home visit. A total of 2750 eligible subjects were invited, and 1347 of them accepted our invitation with an overall response rate of 49.0% at the first wave of data collection in 2009. A total of 1078 subjects were followed up with a follow-up rate of 81.3% at the second wave of data collection in 2010. A total of 617 participants returned for an overall follow-up rate of 57.2% at the third wave of data collection in 2018 after excluding participants who died during the follow-up period. All subjects underwent face-to-face interviews and physical examinations. Data were collected using a standardized questionnaire and health checkup. The flowchart for the recruitment procedures of study subjects is presented in Supplementary Fig. S1. The final sample included 626 older adults with at least two repeated measurements across the 10-year follow-up period. The mean age of study subjects was 75.75 years and 47.12% of them were women.

### Measurements

#### Sociodemographic factors, lifestyle behaviors, and disease histories

The standardized questionnaire comprises sociodemographic characteristics, educational attainment levels, marital statuses, income levels, smoking habits, habitual alcohol intake levels, leisure-time physical activities, and personal histories of diagnosed hypertension; diabetes mellitus; heart disease; hyperlipidemia; stroke; and cancer, including current anti-diabetes, hypertension, heart, and hyperlipidemia medications. Persons who self-reported smoking, alcohol drinking or physical activity were classified into the group with their specific characteristics.

#### Anthropometric measurements

The anthropometric measurements include body height, weight, BMI, WC, hip circumference (HC), and WHR. Weight was measured in kilograms to the nearest 0.5 kg with an electronic medical scale (seca, Hamburg, Germany). Height in meters was measured to the nearest 0.5 cm with a fixed stadiometer (seca). BMI was calculated as weight in kilograms divided by the square of height in meters (kg/m^2^). WC was measured midway point between the inferior margin of the last rib and the crest of ilium in a horizontal plane when the participant was standing. HC was taken as the distance around the pelvis at the point of maximal protrusion of the buttocks. WC and HC were measured to the nearest 0.1 kg, and WHR was then calculated. An electronic device was used to measure blood pressure in a seated position (COLIN, VP-1000, Japan).

#### Body composition

DXA (Lunar DPX, General Electric, Madison, WI, USA) was used to determine the whole body and regional distributions of fat and lean mass. The participants lied in a supine position, clad in their underwear, without any metal items in their clothing or elsewhere. The operator performed a whole-body scan on each participant. The whole-body composition analysis provided data on different regions, such as spine, neck, arms, legs, and trunk. DXA analysis software (Lunar enCORE version 8.60.006; General Electric) analyzed the lean soft tissue mass and the fat mass in the arms, legs, trunk, and the entire body. The equipment was calibrated using a standardized phantom each day. The software automatically located the outer and inner margins of the abdominal wall on both sides of the projected DXA image based on the fat and lean mass profiles across the abdomen at the level of the fourth lumbar vertebrae. The software estimated the total fat mass in the abdominal cavity, a region that included subcutaneous and visceral fats [22]. In addition, the DXA estimation of AF showed high correlations with corresponding computerized tomography (CT) scan (*r* = 0.87 in men; *r* = 0.98 in women) [23] or magnetic resonance imaging (MRI) (*r* = 0.98) [24]. DXA is a good alternative to CT or MRI for predicting total abdominal fat. It is widely available, relatively inexpensive, and has relatively low radiation exposure.

#### Frailty status

Frailty is defined on the basis of well-established, standardized, and widely accepted phenotype described by Fried et al. [25]. It comprises five components: shrinking, slowness, weakness, poor endurance and energy, and low physical activity level. Shrinking refers to elders who had unintentional weight loss of ≥3 kg in the prior year. Slowness is determined by the first quintile of the time needed to walk 15 ft based on gender and height subgroups [25]. Weakness is defined as grip strength in the lowest quintile based on gender and BMI subgroups [25]. Poor endurance and energy is measured by self-reported exhaustion, identified by two questions from the Center for Epidemiological Studies Depression Scale [26] . Low physical activity level is measured by a weighted score of kilocalories expended per week based on each participant’s self-report. Those with none of the above components are considered robust, whereas those with more than two components are regarded as frail.

#### Cognitive function assessment

MMSE scale is widely used to assess cognitive function in older adults*.* MMSE is developed by Folstein M. F. and Folstein S. E [27]. It is initially designed for the grading evaluation of patients with cognitive impairment and now has become a widely used test to screen for cognitive disorders in epidemiological studies and assess cognitive changes in clinical trials. The MMSE test includes simple questions and problems in multiple domains of cognitive function: orientation (time and place of the test, maximum score of 5 each for time and place with a total of 10 points), registration (repeating lists of words, maximum score of 3), attention and calculation (arithmetic such as serial sevens, maximum score of 5), language use (naming a pencil and a watch) and comprehension (maximum score of 8), registration recall (maximum score of 3), and basic motor skills (maximum score of 1). The total score is 30, and the cut-point score varies with different educational levels. An elder is considered to have cognitive impairment if the total MMSE score is less than 27, 24, and 21, with educational attainment of more than 6 years, equal or less than 6 years, and illiteracy, respectively. The cut-points are determined through a modification from literature in the Chinese and Korean versions of MMSE [28, 29].

### Statistical analysis

Summary statistics for the baseline variables were shown as frequency (proportion), assessed through the chi-square test and Fisher’s exact test. For analyzing patterns of BMI, FM, WC, WHR, and AF trajectories, we used multilevel models and performed a cluster analysis. Multilevel models were used to estimate the parameters of individual growth curves, including intercept, and regression coefficients of linear and quadratic terms. The interaction term between covariates and linear and quadratic terms of time was used to determine the covariates that modified obesity marker trajectories. Then, individuals’ parameter estimates were entered into the cluster analysis, using Ward’s method and cubic clustering criterion to identify the patterns by classifying these elders into cluster groups according to their parameter estimates. Finally, multivariate logistic regression analyses were performed to determine whether individuals at different cluster groups of BMI, FM, WC, WHR, and AF trajectories are associated with cognitive impairment and cognitive decline. To adjust for covariates, multivariate models were built by following the guideline proposed by Hosmer and Lemeshow (2013) [30]. Step 1, we built a univariate model for cluster groups of BMI, WC, WHR, FM, and VF trajectories along with age and sex. Step 2, we built a multivariate model 1 by considering significant sociodemographic factors (education level and marital status) and lifestyle behaviors (smoking, alcohol drinking, and physical activity) identified in the univariate model. Step 3, multivariate model 2 is additionally adjusted for significant factors of disease history, fall history, sleep disturbance, frailty status, and baseline cognitive impairment identified in univariate models. Then, the joint associations of obesity markers (BMI, WHR, WC, FM or AF) with dependent variables (cognitive impairment and cognitive decline) were explored by calculating odds ratios (ORs) and 95% confidence intervals (CIs) after adjusting for age, sex, and multiple variables. The obesity marker trajectory cluster with the lowest likelihood of cognitive impairment or change was treated as reference group. When joint effects of these obesity marker trajectories were explored, the clusters with similar odds ratios were grouped into one. All analyses were performed with SAS version 9.4 (SAS, Cary, NC). All *p*-values are two-tailed, and a *p*-value < 0.05 is considered statistically significant.

## Results

**Fig. 1:**
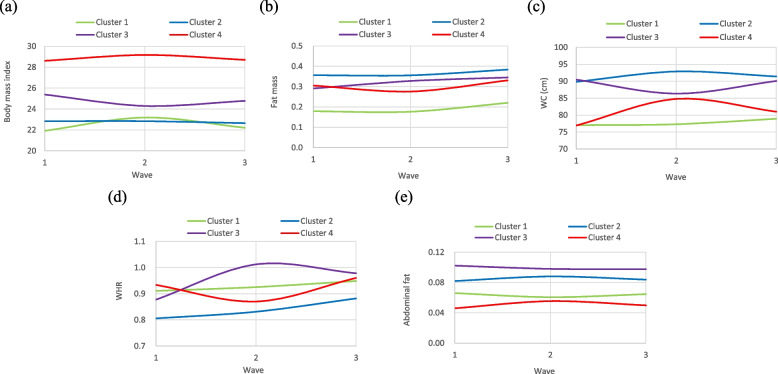
The three trajectory patterns of **a** body mass index, **b** fat mass, **c** WC, **d** WHR, and **e** abdominal fat in the 10-years follow-up

Of the 626 older adults who participated in the study, 168 had incident cognitive impairment and 156 had cognitive decline higher than the 75th percentile during the 10-year follow-up period. The baseline characteristics according to those with and without cognitive impairment and cognitive decline higher and lower than (or equal to) the 75th percentile are provided in Table 1. Older adults with cognitive impairment had significantly higher proportions of age groups 75–84 years and ≥ 85 years (*p* < 0.001), unmarried (*p* = 0.03), smoking (*p* = 0.02), hypertension (*p* = 0.04), diabetes mellitus (*p* = 0.01), stroke (*p* = 0.001), and frailty status (*p* = 0.002) than those without cognitive impairment. We also observed significantly higher proportions of age group ≥85 years (*p* = 0.03), no education or received primary education (*p* = 0.01), diabetes mellitus (*p* = 0.005), and frailty status (*p* = 0.03) in older adults with cognitive decline higher than the 75th percentile. Obesity marker trajectory patterns, including BMI, FM, WC, WHR, and AF, are profiled in Fig. 1. The comparisons of incident cognitive impairment and cognitive decline among four patterns of BMI, FM, WC, WHR, and AF trajectories are summarized in Table 2. The incidence rates of cognitive impairment were significantly different among older adults in patterns of FM trajectories (i.e., consistently low-level, consistently high-level, elevated with median-level, and flat U-shaped with median-level groups) and AF trajectories (i.e., low with flat inverse U-shaped, median with flat inverse U-shaped, consistently high-level, and low with flat U-shaped groups). As for cognitive decline, we observed differences among patterns of WHR trajectories (i.e., high level with increasing trend, low level with slightly increasing trend, high level with increasing–decreasing trend, and high level with U-shaped groups).

**Table 1:** Comparisons of baseline socio-demographic factors, lifestyle behaviors, disease history, and frailty status according to cognitive impairment and cognitive decline

Variables	Cognitive impairment n (%)			Cognitive decline (> 75 Pctl) n (%)		
	No (*n* = 458)	Yes (*n* = 168)	*p* value	No (*n* = 470)	Yes (*n* = 156)	*p* value
***Socio-demographic factors***						
Age (years)			< 0.001			0.03
65–74	319 (69.65)	82 (48.81)		304 (64.68)	97 (62.18)	
75–84	131 (28.6)	66 (39.29)		151 (32.13)	46 (29.49)	
≥ 85	8 (1.75)	20 (11.9)		15 (3.19)	13 (8.33)	
Sex			0.05			1.00
Men	231 (50.44)	100 (59.52)		249 (52.98)	82 (52.56)	
Women	227 (49.56)	68 (40.48)		221 (47.02)	74 (47.44)	
Education			0.13			0.01
No education	145 (31.66)	66 (39.29)		150 (31.91)	61 (39.10)	
Primary education	177 (38.65)	52 (30.95)		166 (35.32)	63 (40.38)	
Secondary or tertiary education	136 (29.69)	50 (29.76)		154 (32.77)	32 (20.51)	
Married			0.03			0.15
No	111 (24.24)	56 (33.33)		118 (25.11)	49 (31.41)	
Yes	347 (75.76)	112 (66.67)		352 (74.89)	107 (68.59)	
***Lifestyle behaviors***						
Smoking	28 (6.11)	20 (11.90)	0.02	33 (7.02)	15 (9.62)	0.38
Alcohol drinking	61 (13.32)	28 (16.67)	0.35	69 (14.68)	20 (12.82)	0.66
Physical activity	354 (77.29)	121 (72.02)	0.21	365 (77.66)	110 (70.51)	0.09
***Disease history***						
Hypertension	222 (48.47)	98 (58.33)	0.04	236 (50.21)	84 (53.85)	0.49
Diabetes mellitus	71 (15.5)	41 (24.40)	0.01	72 (15.32)	40 (25.64)	0.005
Heart disease	124 (27.07)	47 (27.98)	0.90	126 (26.81)	45 (28.85)	0.70
Hyperlipidemia	126 (27.51)	33 (19.64)	0.06	119 (25.32)	40 (25.64)	1.00
Stroke	18 (3.93)	19 (11.31)	0.001	23 (4.89)	14 (8.97)	0.09
Cancer	28 (6.11)	11 (6.55)	0.99	30 (6.38)	9 (5.77)	0.93
Fall history	80 (17.47)	39 (23.21)	0.13	92 (19.57)	27 (17.31)	0.61
Sleep disturbance	205 (44.76)	80 (47.62)	0.59	214 (45.53)	71 (45.51)	1.00
***Frailty status***			0.002			0.03
No	444 (96.94)	152 (90.48)		453 (96.38)	143 (91.67)	
Yes	14 (3.06)	16 (9.52)		17 (3.62)	13 (8.33)

**Table 2:** Comparisons of pattern of body mass index, fat mass, waist, WHR and abdominal fat trajectories according to cognitive impairment and cognitive decline

Variables	Cognitive impairment n (%)			Cognitive decline (> 75 Pctl) n (%)		
	No (*n* = 458)	Yes (*n* = 168)	*p* value	No (*n* = 470)	Yes (*n* = 156)	*p* value
BMI trajectories			0.13			0.78
Cluster 1: very low level with inverse U-shaped trend group	61 (63.54)	35 (36.46)		69 (71.88)	27 (28.13)	
Cluster 2: consistently low-level group	163 (74.43)	56 (25.57)		167 (76.26)	52 (23.74)	
Cluster 3: median level with flat U-shaped trend group	160 (74.42)	55 (25.58)		164 (76.28)	51 (23.72)	
Cluster 4: high-level group	74 (77.08)	22 (22.92)		70 (72.92)	26 (27.08)	
Fat mass trajectories			0.008			0.26
Cluster 1: consistently low-level group	41 (59.42)	28 (40.58)		54 (78.26)	15 (21.74)	
Cluster 2: consistently high-level group	148 (80.43)	36 (19.57)		144 (78.26)	40 (21.74)	
Cluster 3: elevated with median level group	130 (72.63)	49 (27.37)		125 (69.83)	54 (30.17)	
Cluster 4: flat U-shaped in median level group	139 (71.65)	55 (28.35)		147 (75.77)	47 (24.23)	
WC trajectories			0.46			0.31
Cluster 1: low level with slightly increasing group	98 (71.53)	39 (28.47)		105 (76.64)	32 (23.36)	
Cluster 2: high level with flat inverse U-shaped group	117 (74.05)	41 (25.95)		110 (69.62)	48 (30.38)	
Cluster 3: high level with flat U-shaped group	152 (70.7)	63 (29.30)		164 (76.28)	51 (23.72)	
Cluster 4: low level with flat inverse U-shaped group	91 (78.45)	25 (21.55)		91 (78.45)	25 (21.55)	
WHR trajectories			0.09			0.03
Cluster 1: high level with increasing trend group	186 (68.13)	87 (31.87)		200 (73.26)	73 (26.74)	
Cluster 2: low level with slightly increasing trend group	161 (77.78)	46 (22.22)		169 (81.64)	38 (18.36)	
Cluster 3: high level with increasing-decreasing trend group	48 (75.00)	16 (25.00)		42 (65.63)	22 (34.38)	
Cluster 4: high level with U-shaped group	63 (76.83)	19 (23.17)		59 (71.95)	23 (28.05)	
Abdominal fat trajectories			< 0.001			0.51
Cluster 1: low with flat inverse U-shaped group	97 (61.39)	61 (38.61)		117 (74.05)	41 (25.95)	
Cluster 2: median with flat inverse U-shaped group	138 (75.41)	45 (24.59)		132 (72.13)	51 (27.87)	
Cluster 3: consistently high-level group	175 (80.28)	43 (19.72)		167 (76.61)	51 (23.39)	
Cluster 4: low with flat U-shaped group	48 (71.64)	19 (28.36)		54 (80.60)	13 (19.40)	

*BMI* Body mass index, *WC* Waist circumference, *WHR* Waist-to-hip ratio

The ORs of cognitive impairment and cognitive decline for obesity marker trajectory patterns are presented in Table 3. Groups from FM, WHR, and AF trajectories were significantly associated with cognitive impairment, whereas WHR was the only significant predictor of cognitive decline. After multivariate adjustment, the adjusted OR (95% CI) of cognitive impairment for FM trajectory in Cluster 2 was 0.41 (0.22, 0.85); for WHR trajectory in Cluster 4 was 0.43 (0.22, 0.84); and for AF trajectory in Clusters 2, 3, and 4 were 0.44 (0.26, 0.77), 0.33 (0.18, 0.61), and 0.39 (0.18, 0.82), respectively, compared with their counterparts in Cluster 1. For cognitive decline, the adjusted OR for WHR trajectory in Cluster 2 was 0.43 (0.22, 0.85), compared with that for WHR trajectory in Cluster 3.

**Table 3:** Odds Ratio and 95% confidence intervals for body mass index, fat mass, waist, WHR and abdominal fat trajectories with cognitive impairment and cognitive decline

		Cognitive impairment OR (95% CI)			Cognitive decline (> 75 Pctl) OR (95% CI)		
	*n*	Age and sex adjusted model	Multivariate model ^1^	Multivariate model ^2^	Age and sex adjusted model	Multivariate model ^1^	Multivariate model ^2^
BMI trajectories							
Cluster 1	96	1.00	1.00	1.00	1.00	1.00	1.00
Cluster 2	219	0.59 (0.35, 1.01)	0.58 (0.34, 1.01)	0.70 (0.38, 1.29)	0.85 (0.49, 1.46)	0.84 (0.48, 1.46)	0.87 (0.49, 1.55)
Cluster 3	215	0.55 (0.32, 0.94)*	0.54 (0.31, 0.94)*	0.59 (0.32, 1.09)	0.83 (0.48, 1.45)	0.84 (0.48, 1.48)	0.86 (0.48, 1.55)
Cluster 4	96	0.52 (0.27, 0.99)*	0.49 (0.25, 0.95)*	0.65 (0.31, 1.34)	0.99 (0.52, 1.87)	0.98 (0.51, 1.89)	0.98 (0.50, 1.93)
Fat mass trajectories							
Cluster 1	69	1.00	1.00	1.00	0.67 (0.34, 1.31)	0.65 (0.32, 1.30)	0.63 (0.31, 1.29)
Cluster 2	184	0.35 (0.18, 0.68)**	0.37 (0.19, 0.71)**	0.41 (0.20, 0.85)*	0.65 (0.40, 1.05)	0.68 (0.42, 1.11)	0.65 (0.39, 1.08)
Cluster 3	179	0.54 (0.29, 1.01)	0.56 (0.30, 1.06)	0.54 (0.27, 1.08)	1.00	1.00	1.00
Cluster 4	194	0.57 (0.31, 1.04)	0.58 (0.31, 1.07)	0.65 (0.33, 1.28)	0.76 (0.48, 1.21)	0.77 (0.48, 1.23)	0.79 (0.49, 1.27)
WC trajectories							
Cluster 1	137	1.00	1.00	1.00	0.73 (0.42, 1.26)	0.68 (0.39, 1.19)	0.68 (0.38, 1.21)
Cluster 2	158	0.68 (0.39, 1.17)	0.67 (0.39, 1.18)	0.59 (0.32, 1.09)	1.00	1.00	1.00
Cluster 3	215	0.77 (0.47, 1.27)	0.77 (0.46, 1.28)	0.63 (0.36, 1.13)	0.71 (0.45, 1.14)	0.71 (0.44, 1.14)	0.70 (0.43, 1.13)
Cluster 4	116	0.64 (0.35, 1.17)	0.66 (0.36, 1.21)	0.53 (0.27, 1.04)	0.60 (0.34, 1.07)	0.57 (0.31, 1.02)	0.61 (0.33, 1.13)
WHR trajectories							
Cluster 1	273	1.00	1.00	1.00	0.74 (0.41, 1.34)	0.78 (0.43, 1.43)	0.70 (0.38, 1.30)
Cluster 2	207	0.68 (0.43, 1.08)	0.72 (0.45, 1.15)	0.70 (0.41, 1.18)	0.41 (0.22, 0.78)**	0.45 (0.24, 0.87)*	0.43 (0.22, 0.85)*
Cluster 3	64	0.68 (0.35, 1.30)	0.70 (0.36, 1.35)	0.60 (0.29, 1.25)	1.00	1.00	1.00
Cluster 4	82	0.54 (0.30, 0.98)*	0.55 (0.30, 1.02)	0.43 (0.22, 0.84)*	0.78 (0.38, 1.59)	0.84 (0.41, 1.75)	0.75 (0.35, 1.58)
Abdominal fat trajectories							
Cluster 1	158	1.00	1.00	1.00	0.98 (0.59, 1.61)	0.94 (0.57, 1.57)	0.89 (0.53, 1.50)
Cluster 2	183	0.47 (0.29, 0.78)**	0.50 (0.30, 0.83)**	0.44 (0.26, 0.77)**	1.00	1.00	1.00
Cluster 3	218	0.37 (0.22, 0.65)***	0.39 (0.23, 0.69)**	0.33 (0.18, 0.61)***	0.81 (0.50, 1.30)	0.87 (0.53, 1.41)	0.83 (0.51, 1.37)
Cluster 4	67	0.60 (0.31, 1.15)	0.55 (0.28, 1.06)	0.39 (0.18, 0.82)*	0.63 (0.31, 1.27)	0.59 (0.28, 1.21)	0.61 (0.29, 1.27)

*BMI* Body mass index, *WC* Waist circumference, *WHR* Waist-to-hip ratio; *: *p* < 0.05; **: *p* < 0.01; ***: *p* < 0.001

^1^Multivariate adjustment for age, sex, exercising program, education, marital status, BMI, smoking, alcohol drinking, and physical activity

^2^Multivariate adjustment for age, sex, exercising program, education, marital status, BMI, smoking, alcohol drinking, physical activity, hypertension, diabetes mellitus, heart disease, hyperlipidemia, stroke, cancer, fall history, sleep disturbance, frailty and baseline cognitive status

**Fig. 2:**
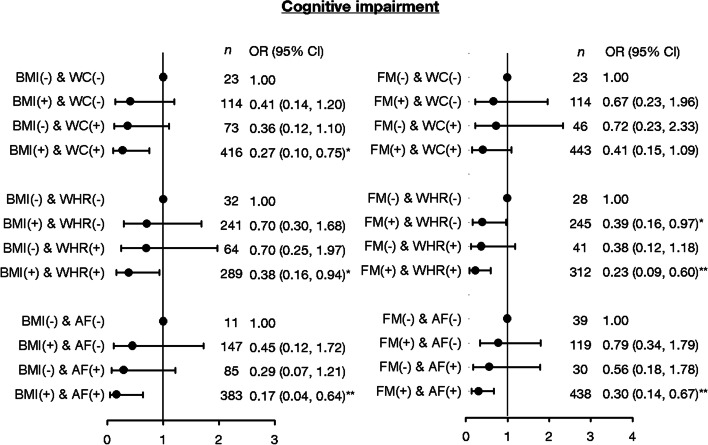
Joint relationship of overall obesity markers (BMI or FM) and central obesity markers (WC, WHR or AF) for cognitive impairment. Symbol “-“ presents cluster 1, and symbol “+” presents clusters 2–4 for BMI, FM, WC, WHR and AF trajectories

**Fig. 3:**
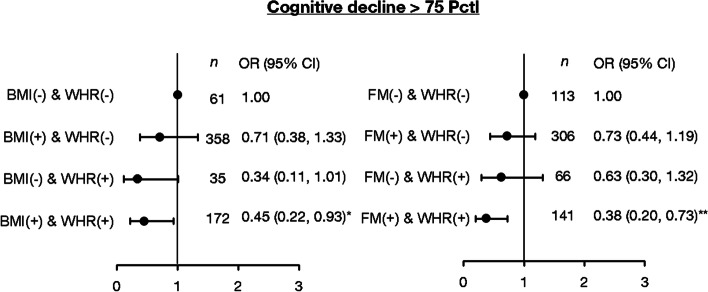
Joint relationship of overall obesity markers (BMI or FM) and central obesity markers (WHR) for cognitive decline. BMI trajectories: - (cluster 1) and + (cluster 2–4). FM trajectories: - (cluster 3) and + (cluster 1, 2, 4). WHR trajectories: - (cluster 1,3,4) and + (cluster 2)

To explore the joint effects of these obesity marker trajectories, categories with similar effects were clumped into one. Consistently low-level, median level with flat U-shaped trend, and high-level groups from BMI trajectory and high level with flat inverse U-shaped, high level with flat U-shaped, and low level with flat inverse U-shaped groups from WC trajectory were bundled. Moreover, low level with slightly increasing trend, high level with increasing–decreasing trend, and high level with U-shaped groups from WHR trajectory and median with flat inverse U-shaped, consistently high-level, and low with flat U-shaped groups from AF trajectory were clumped together. The joint associations of overall obesity markers and central obesity markers for cognitive impairment and cognitive decline are illustrated in Figs. 2 and 3. The overall obesity defined by BMI or FM and central obesity measures, such as WC, WHR, and AF, had combined effects on the incidence of cognition impairment (BMI & WC: ORs = 0.27; BMI & WHR: ORs = 0.38; BMI & AF: ORs = 0.17; FM & WC: ORs = 0.41, FM & WHR: ORs = 0.23, and FM & AF: ORs = 0.30). In addition, the BMI/FM-based overall obesity combined with WHR-based central obesity were significantly associated with cognitive decline (BMI & WHR: ORs = 0.45; FM & WHR: ORs = 0.38). We observed greater magnitude in these joint associations than that in independent associations, except for the joint association of BMI and WHR for cognitive decline.

## Discussion

Our study is the first to examine the effects of BMI, FM, WC, WHR, and AF trajectories on cognitive impairment and cognitive decline in older adults. Our results indicate that the consistently high-level group from FM trajectory and the consistently high-level, median with flat inverse U-shaped, and low with flat U-shaped groups from AF trajectory were associated with reduced risks of cognitive impairment. Moreover, the low with slightly increasing trend group from WHR trajectory had low risks of cognitive impairment and cognitive decline. These findings are consistent with our expectation that trajectories with consistently high levels or increasing trends are associated with a lower likelihood of cognitive impairment or cognitive decline. Although BMI and WC trajectory patterns were unassociated with risks of cognitive impairment or cognitive decline in older adults, the joint effects of the relatively high-level groups from BMI and WC trajectories; relatively high fluctuation groups from WHR trajectory; and relatively low-level groups from AF trajectory were associated with the smallest OR of cognitive impairment in older adults, suggesting that maintaining overall obesity (high BMI or FM) but low WHR or AF in older adults prevents cognitive impairment or cognitive decline incidence.

Obesity is correlated with inflammation [31], hyperinsulinemia/insulin resistance [32], gut dysbiosis [33], and systemic mediators [31], which have been consistently associated with increased risks of dementia [34]. Pathophysiological processes of dementia begin many years prior to detectable cognitive changes and perhaps decades before the onset of clinical symptoms [35, 36] and can lead to weight loss before the clinical onset of dementia [37]. However, obesity seems to play different roles in dementia at different life stages [38]. Mid-life obesity has been reported to be a risk factor for dementia or cognitive decline [14], but the association between late-life obesity and dementia is inconclusive [39].

Obesity markers are dynamic and fluctuate over time, but recent studies have focused on the relationship between obesity markers and cognitive function using a cross-sectional design [7–19]. Only one prior study examined the associations between obesity markers measured at one time point and cognitive impairment or cognitive decline using a longitudinal study design [14]. Our study assessed the associations of obesity trajectories with cognitive impairment and cognitive decline in older people and found that relatively high-level trajectory patterns of overall obesity markers were associated with lower risks of incident cognitive impairment and cognitive decline, consistent with previous results that higher BMI [7–12, 21], FM [17, 19], WC [21], and AF [16] and lower WHR [12–15] reduced risks of cognitive impairment. A prior study reported that older women with low BMI were more likely to have MCI, but older men with elevated BMI were more likely to have MCI [11]. Similarly, our study found a significant interaction between gender and WHR trajectory on the risks of cognitive impairment (*p* = 0.04). Compared with the high level with increasing trend group from WHR trajectory, the high level with U-shaped group was associated with lower risks of cognitive impairment in older men (OR = 0.30, 95% CI = 0.13–0.71; *p* = 0.006), but not in older women (OR = 1.03, 95% CI = 0.31–3.43; *p* = 0.96). Due to the existence of gender differences in body composition, exploring the gender effect on the associations between obesity markers and cognitive function in future research is needed.

Although the trajectory of obesity markers can reveal what patterns of change were associated with cognitive impairment or decline, the temporal relationship is not clear because the time points for measuring obesity markers overlapped with those for cognitive function assessment. In order to have clear-cut time sequence of changes in obesity markers and subsequent cognitive function status or decline, we further explored the relationships of changes in obesity markers between baseline and the first year with cognitive impairment at endpoint or cognitive decline during the period of the first and 10 years (Supplementary Table S1). Based on baseline and baseline-first year changes in obesity markers, study subjects were grouped into four groups **(**low at baseline and slow change, low at baseline but rapid change, high at baseline but slow change, and high at baseline and rapid change). Significant associations between obesity groups and subsequent cognitive impairment were observed for waist, WHR, and abdominal fat, which provided the evidence of temporal relationship.

The protective effect of overall obesity on outcomes is referred to as the “obesity paradox.” Many epidemiologic evidence supports the hypothesis that old age obesity is linked to favorable outcomes, such as delay in cognitive decline [7, 9, 10, 12, 40]. Hormone leptin is recognized to explain the mechanism for this protective effect [34]. Leptin is a circulating hormone produced by adipose tissue, which may play as a cognitive enhancer that facilitates learning and memory performance by regulating hippocampal synaptic plasticity and amyloid β-processing [41, 42]. The other possible hypothesis is the “survival effect”. Persons who are susceptible to the negative effects of obesity may die earlier, and those who survive into old age may be more likely to resistant to negative effects of obesity effects. In the present study, the median level with flat U-shaped trend and high-level groups from BMI trajectory were significantly associated with lower risks of cognitive impairment, but their effects became non-significant after adjusting for the comorbidities and baseline cognitive function. When overall obesity measured by FM, consistently high-level group was associated with a 59% reduction in risk of incident cognitive impairment after considering all covariates.

Overall obesity is commonly defined by BMI, which is highly related to percentage body fat and total body fat [43]. However, BMI does not consider differences in body composition and body fat contribution to overall. Abdominal obesity, a key component of central obesity, refers to the presence of excess fat in the abdominal area, which can be obtained using simple measures, such as WC or WHR [44]. Our study is the first to report the joint effects of overall and abdominal obesities on cognitive function or cognitive decline. These findings suggest that overall and abdominal obesities should be combined to predict cognitive impairment and decline in older adults for interpreting extra variations that cannot be explained by the use of overall or central obesity indicators alone.

This investigation is an observational, prospective, community-based cohort study that included 626 participants aged 65 or older with repeated measures of obesity markers and cognitive function over a 10-year follow-up period. In the period of data collection process, valid and reliable measurement instruments with standardized protocols were used. Given that education level was correlated with cognitive status assessment, cognitive impairment was defined according to MMSE scores and education levels to adjust for bias arising from variations in education attainment. However, some limitations should be considered in evaluating our results. First, we measured FM and AF using DXA, which is less accurate than CT scan or MRI results. However, previous studies that measured body fat using DXA obtained comparable accuracy compared with that using CT and MRI [23, 24]. In addition, DXA is accessible and has practical advantage because it is a low-dose radiation technique compared with CT and MRI, which are costly and require trained professionals for specific post-processing and analyzing examinations. Due to this limitation, the widespread use of CT and MRI in daily routine is constrained. Second, previous studies found sex differences in the effects of BMI and FM on cognitive impairment [11, 17], but our sample size is not large enough to observe such differences. Third, all our study subjects are community-dwelling men and women aged 65 or older from Taichung City, which can reduce the generalizability of our results. Last, the data were collected at baseline, and one-year and 10-year follow-up. Elders were included for those who had at least two repeated measurements across the 10-year follow-up period. Due to the limited time points for measuring obesity markers, the estimates of the 10-year trajectory pattern may not be accurate. However, due to few prior longitudinal studies, our study’s findings may provide new insight to this line of research question. Future studies are warranted to validate the results.

## Conclusion

This study demonstrates that FM, WHR, and AF trajectories are associated with incident cognitive impairment, and WHR trajectory is a key predictor for the cognitive decline in older adults. Our findings suggest that FM and AF trajectories with consistent high levels and WHR trajectory with high level with U-shaped group are associated with low risks of incident cognitive impairment in older adults. Similarly, WHR trajectory with low but slowly increasing trend is associated with a decreased risk of cognitive decline. Our findings may be useful in screening those who are at high risks of incident cognitive impairment or cognitive decline and in tailoring interventions for cognitive decline prevention.

## Supplementary Information

**Additional file 1: Supplementary Fig. S1.** Time points of the data collection for the TCHS-E. **Supplementary Table S1.** Odds Ratio and 95% confidence intervals for subgroups of baseline and changes in body mass index, fat mass, waist, WHR and abdominal fat between baseline and first year with cognitive impairment at endpoint and cognitive decline between first year and endpoint.

## Data Availability

The datasets generated and/or analyzed during the current study are not publicly available due to the policy declared by Ministry of Health and Welfare in Taiwan but are available from the corresponding author on reasonable request.

## Abbreviations

MCI: Mild cognitive impairment
BMI: Body mass index
FM: Fat mass
WC: Waist circumference
WHR: Waist-to-hip ratio
AF: Abdominal fat
DXA: Dual-energy X-ray absorptiometry
BIA: Bioimpedance analysis
MMSE: Mini-Mental State Examination
TCHS-E: Taichung Community Health Study for Elders
HC: Hip circumference
CT: Computerized tomography
MRI: Magnetic resonance imaging
ORs: Odds ratios
CIs: Confidence intervals
