# Supplementary material for: Obesity marker trajectories and cognitive impairment in older adults: a 10-year follow-up in Taichung community health study for elders
Source: BMC Psychiatry. 2022 Nov 30;22:748. doi: 10.1186/s12888-022-04420-1 (PMC9710179; doi:10.1186/s12888-022-04420-1)
Supplement: Supplementary file 1 — Additional file 1: Supplementary Fig. S1. Time points of the data collection for the TCHS-E. Supplementary Table S1. Odds Ratio and 95% confidence intervals for subgroups of baseline and changes in body mass index, fat mass, waist, WHR and abdominal fat between baseline and first year with cognitive impairment at endpoint and cognitive decline between first year and endpoint. [file 12888_2022_4420_MOESM1_ESM.docx]

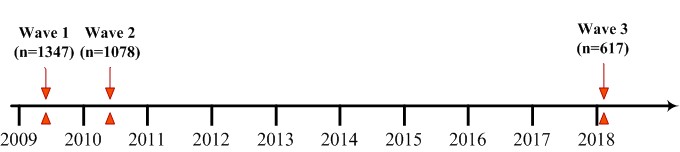


**Supplementary Fig. S1.** Time points of the data collection for the TCHS-E

**Supplementary Table S1.** Odds Ratio and 95% confidence intervals for subgroups of baseline and changes in body mass index, fat mass, waist, WHR and abdominal fat between baseline and first year with cognitive impairment at endpoint and cognitive decline between first year and endpoint.

|  |  | Cognitive impairment at W3 OR (95% CI) | | |  | | Cognitive decline (W3-W2) (>75 Pctl) OR (95% CI) | | | |  |
| --- | --- | --- | --- | --- | --- | --- | --- | --- | --- | --- | --- |
|  | *n* | Age and sex adjusted model | Multivariate model 1 | Multivariate model 2 | |  | | Age and sex adjusted model | Multivariate model 1 | Multivariate model 2 | |
| BMI |  |  |  |  | |  | |  |  |  | |
| Group 1 | 163 | 1.00 | 1.00 | 1.00 | |  | | 1.00 | 1.00 | 1.00 | |
| Group 2 | 150 | 1.25 (0.75, 2.10) | 1.24 (0.73, 2.09) | 1.49 (0.83, 2.70) | |  | | 1.17 (0.70, 1.97) | 1.14 (0.67, 1.92) | 1.22 (0.71, 2.12) | |
| Group 3 | 150 | 1.06 (0.63, 1.79) | 1.03 (0.60, 1.77) | 1.34 (0.73, 2.45) | |  | | 1.03 (0.61, 1.74) | 1.03 (0.60, 1.77) | 1.14 (0.65, 2.02) | |
| Group 4 | 163 | 0.91 (0.54, 1.53) | 0.93 (0.55, 1.57) | 0.97 (0.53, 1.77) | |  | | 0.92 (0.55, 1.54) | 0.93 (0.55, 1.57) | 0.93 (0.53, 1.63) | |
| Fat mass |  |  |  |  | |  | |  |  |  | |
| Group 1 | 170 | 1.00 | 1.00 | 1.00 | |  | | 1.00 | 1.00 | 1.00 | |
| Group 2 | 143 | 1.30 (0.79, 2.13) | 1.30 (0.78, 2.16) | 1.56 (0.90, 2.70) | |  | | 1.33 (0.81, 2.20) | 1.34 (0.81, 2.24) | 1.47 (0.86, 2.50) | |
| Group 3 | 143 | 0.68 (0.37, 1.26) | 0.72 (0.39, 1.34) | 0.71 (0.35, 1.41) | |  | | 0.89 (0.48, 1.64) | 0.96 (0.52, 1.78) | 1.01 (0.53, 1.94) | |
| Group 4 | 170 | 0.77 (0.43, 1.38) | 0.82 (0.46, 1.48) | 0.90 (0.47, 1.75) | |  | | 1.08 (0.61, 1.92) | 1.17 (0.65, 2.11) | 1.26 (0.68, 2.00) | |
| WC |  |  |  |  | |  | |  |  |  | |
| Group 1 | 175 | 1.00 | 1.00 | 1.00 | |  | | 1.00 | 1.00 | 1.00 | |
| Group 2 | 137 | 1.71 (0.97, 3.01) | 1.65 (0.93, 2.93) | 1.71 (0.91, 3.20) | |  | | 1.58 (0.93, 2.70) | 1.53 (0.89, 2.63) | 1.61 (0.91, 2.83) | |
| Group 3 | 127 | 2.09 (1.19, 3.67)* | 1.92 (1.09, 3.40)* | 1.97 (1.06, 3.67)* | |  | | 1.46 (0.83, 2.55) | 1.44 (0.81, 2.56) | 1.55 (0.85, 2.82) | |
| Group 4 | 187 | 1.86 (1.10, 3.15)* | 1.80 (1.06, 3.05)* | 1.64 (0.91, 2.94) | |  | | 1.06 (0.62, 1.80) | 1.00 (0.58, 1.71) | 1.02 (0.58, 1.80) | |
| WHR |  |  |  |  | |  | |  |  |  | |
| Group 1 | 187 | 1.00 | 1.00 | 1.00 | |  | | 1.00 | 1.00 | 1.00 | |
| Group 2 | 126 | 1.71 (0.97, 3.01) | 1.65 (0.93, 2.93) | 1.71 (0.91, 3.20) | |  | | 1.39 (0.80, 2.44) | 1.33 (0.76, 2.35) | 1.35 (0.75, 2.42) | |
| Group 3 | 126 | 2.09 (1.19, 3.67)* | 1.92 (1.09, 3.40)* | 1.97 (1.06, 3.67)* | |  | | 1.81 (1.04, 3.15)* | 1.71 (0.98, 3.00) | 1.71 (0.95, 3.06) | |
| Group 4 | 187 | 1.86 (1.10, 3.15)* | 1.80 (1.06, 3.05)* | 1.64 (0.91, 2.94) | |  | | 1.38 (0.82, 2.32) | 1.34 (0.79, 2.29) | 1.28 (0.74, 2.22) | |
| Abdominal fat |  |  |  |  | |  | |  |  |  | |
| Group 1 | 172 | 1.00 | 1.00 | 1.00 | |  | | 1.00 | 1.00 | 1.00 | |
| Group 2 | 141 | 1.75 (1.06, 2.88)* | 1.70 (1.02, 2.82)* | 2.12 (1.23, 3.68)** | |  | | 1.29 (0.78, 2.13) | 1.27 (0.76, 2.11) | 1.32 (0.78, 2.25) | |
| Group 3 | 141 | 0.79 (0.42, 1.49) | 0.82 (0.44, 1.56) | 1.01 (0.50, 2.02) | |  | | 0.73 (0.38, 1.39) | 0.76 (0.40, 1.46) | 0.82 (0.42, 1.61) | |
| Group 4 | 172 | 0.86 (0.48, 1.55) | 0.89 (0.5, 1.61) | 1.01 (0.53, 1.94) | |  | | 1.23 (0.70, 2.16) | 1.27 (0.72, 2.24) | 1.36 (0.75, 2.46) | |

^1^Multivariate adjustment for age, sex, exercising program, education, marital status, BMI, smoking, alcohol drinking, and physical activity.

^2^Multivariate adjustment for age, sex, exercising program, education, marital status, BMI, smoking, alcohol drinking, physical activity, hypertension, diabetes mellitus, heart disease, hyperlipidemia, stroke, cancer, fall history, sleep disturbance, frailty and baseline cognitive status.

Group 1: low at baseline and slow change (W1<Median & D<Median); group 2: low at baseline but rapid change (W1<Median & D≥Median); group 3: high at baseline but slow change (W1≥Median & D<Median); and group 4: high at baseline and rapid change (W1≥Median & D≥Median).

W1: wave 1; D: difference between baseline and 1^st^ year obesity markers; BMI: body mass index; WC: waist circumference; WHR: waist-to-hip ratio; *: p<0.05.
